# A review of artificial intelligence methods enabled music-evoked EEG emotion recognition and their applications

**DOI:** 10.3389/fnins.2024.1400444

**Published:** 2024-09-04

**Authors:** Yan Su, Yong Liu, Yan Xiao, Jiaqi Ma, Dezhao Li

**Affiliations:** ^1^School of Art, Zhejiang International Studies University, Hangzhou, China; ^2^School of Education, Hangzhou Normal University, Hangzhou, China; ^3^School of Arts and Media, Beijing Normal University, Beijing, China; ^4^College of Science, Zhejiang University of Technology, Hangzhou, China

**Keywords:** music-induced, emotion recognition, artificial intelligence, personalization, applications

## Abstract

Music is an archaic form of emotional expression and arousal that can induce strong emotional experiences in listeners, which has important research and practical value in related fields such as emotion regulation. Among the various emotion recognition methods, the music-evoked emotion recognition method utilizing EEG signals provides real-time and direct brain response data, playing a crucial role in elucidating the neural mechanisms underlying music-induced emotions. Artificial intelligence technology has greatly facilitated the research on the recognition of music-evoked EEG emotions. AI algorithms have ushered in a new era for the extraction of characteristic frequency signals and the identification of novel feature signals. The robust computational capabilities of AI have provided fresh perspectives for the development of innovative quantitative models of emotions, tailored to various emotion recognition paradigms. The discourse surrounding AI algorithms in the context of emotional classification models is gaining momentum, with their applications in music therapy, neuroscience, and social activities increasingly coming under the spotlight. Through an in-depth analysis of the complete process of emotion recognition induced by music through electroencephalography (EEG) signals, we have systematically elucidated the influence of AI on pertinent research issues. This analysis offers a trove of innovative approaches that could pave the way for future research endeavors.

## 1 Introduction

Music serves as a unique medium for people to express their emotions and also can arouse strong emotional responses. Previous studies have shown that the emotional changes induced by appropriate music can relieve listeners’ mental stress (Nawaz et al., 2019; Colin et al., 2023), promote emotional expression ability (Palazzi et al., 2021; Micallef Grimaud and Eerola, 2022; Zhang et al., 2022), improve learning ability (Bergee and Weingarten, 2021; Luo et al., 2023), and so on. Moreover, it also can be applied in the regulation of mood-related disorders such as autism (Carpente et al., 2022; Geretsegger et al., 2022), depression (Geipel et al., 2022; Hartmann et al., 2023), and anxiety (Contreras-Molina et al., 2021; Lu et al., 2021). With the extensive applications of music-induced emotions in medical (Liang et al., 2021), neuroscience (Thaut et al., 2021; Fedotchev et al., 2022), and music retrieval fields (Gomez-Canon et al., 2021), the study of music-induced emotion recognition has received much attention in recent years.

**Figure 1:**
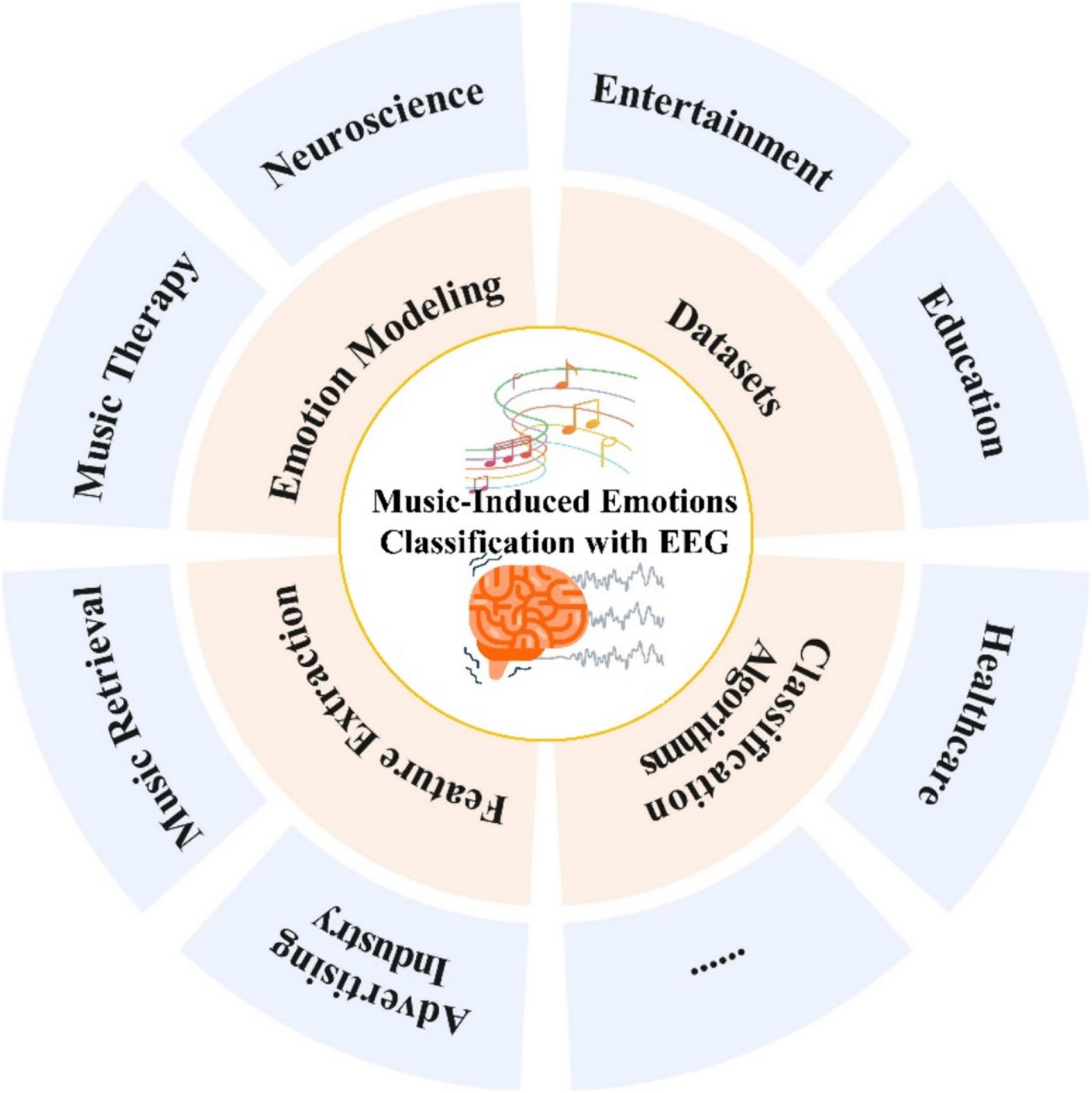
Research and applications of music-induced emotion classification with EEG.

Empirical research on the effects of music on emotions has been discussed for more than three millennia (Perlovsky, 2012), while modern evidence-based work on the effects of music on emotions has its roots in the early 20th century (Humphreys, 1998). Western psychologists and musicians primarily conducted pioneering empirical research on music-induced emotions. A representative example is the experimental research conducted by the American psychologist and music educator Carl Emil Seashore on the emotional expression of music and the emotional impact of music on the listener, combining experiments and psychological tests and proposing the “theory of musical expression,” which emphasizes how elements such as melodies, rhythms, and harmonies of music affect people’s emotional experiences, and lays the foundation for the subsequent development of related work (Metfessel, 1950). With the development of psychology neuroscience and other fields, people gradually realized that the study of music’s induction of emotions also requires an understanding of auditory perception, emotion discrimination, and neural mechanism, which is an interdisciplinary research work (Cui et al., 2022; Ryczkowska, 2022). Musicologists have mainly studied the influence of music on emotion induction from the perspective of different musical features of music (Panda et al., 2023), including analyzing the influence of music on the listener’s emotion from the perspective of musical elements (Ruth and Schramm, 2021), quantitatively analyzing the emotional features of music to find the relationship between the features and emotion (Salakka et al., 2021), developing emotion recognition algorithms based on musical features (Pandey and Seeja, 2022), and exploring cross-cultural emotional understanding of and response to specific musical features (Wang et al., 2022). These studies have made it possible to help people get a better understanding of the relationship between musical features and emotions, and provide theoretical support and practical guidance for the fields of music psychology, music therapy, and creativity, but researchers have also put forward different viewpoints on the individual differences in musical emotional responses and on the objective evaluative validity of emotions as in Figure 1.

With the development of brain science and technology, researchers have found that signals generated by the central nervous system, such as electroencephalography (EEG), magnetoencephalography (MEG), and functional magnetic resonance imaging (MRI) are more objective and reliable in the field of emotion research (Alarcao and Fonseca, 2019; Egger et al., 2019; Saganowski et al., 2023). Among various central nervous system signals, the monitoring of emotions using EEG signals is characterized by the convenience of noninvasive measurements, real-time measurements, and good objectivity. Research on emotion recognition based on EEG signals has been widely used in many disciplines in recent years and has received extensive attention from researchers as in Figure 1. Artificial intelligence (AI) techniques that integrate EEG signals for identifying emotions elicited by music leverage AI’s robust capabilities in data analytics, pattern recognition, and learning, alongside the distinctive benefits of EEG for real-time, non-invasive monitoring of brain activity. AI-enabled EEG recognition of music-induced emotions can accurately and in real-time identify emotions, which has broad applications in many areas including music therapy, education, entertainment, and so on.

How to accurately identify music-induced emotions has always been a difficult research problem due to the subjectivity, abstractness, and individualized differences of music-induced emotions. Researchers have explored a variety of physiological signals to carry out emotion recognition studies, in which using the signal characteristics of facial expressions, researchers have classified emotions including fear, sadness, disgust, surprise, and joy, and the accuracy of obtaining emotion discrimination can be as high as 81% or more, but there are inconsistencies between different cultures in the understanding of facial expressions and the way of expression of facial expressions, which affect the generalizability of the results of the study (Tcherkassof and Dupré, 2021; Witkower et al., 2021). Physiological parameters such as galvanic skin response, heart rate, temperature, blood pressure, and respiration rate have also been utilized for emotion recognition, but these methods are relatively inaccurate for emotion discrimination and highly influenced by other factors (Egger et al., 2019; Saganowski et al., 2023).

In this study, the research methods, processes, and characteristics of EEG in music-induced emotion recognition have been analyzed. The potential future development directions of music-induced emotion based on EEG also have been discussed, which can promote the development of fundamental and application research on music-induced emotion.

## 2 EEG signal and emotions

Measurement of EEG signals is capable of non-invasive, continuous recording of brain activity with a temporal resolution of a few milliseconds. Based on the characteristic waveform signals from different brain regions, EEG signals are widely used in cognitive neuroscience to research emotion regulation and processing, and the results of the related studies provide an important reference for further research on music-induced emotion recognition (Apicella et al., 2022b; Pandey and Seeja, 2022).

### 2.1 EEG signals and acquisition method

The activity of the central nervous system of the brain is closely related to human emotions, mainly realized through electrical communication between neurons (Ahmad et al., 2022). When neurons are stimulated, the membrane potential will rise and fall to form weak electrical pulse signals and emotional changes can be monitored by recording and analyzing EEG signals. EEG signals have the characteristics of small amplitude (10–100 μV), many interference sources, and high uncertainty. To analyze the feature information of EEG, as shown in Table 1, EEG signals are generally classified into δ-band (1–4 Hz), θ-band (4–8 Hz), α-band (8–13 Hz), β-1-band (13–22 Hz), β-2-band (22–30 Hz) and γ-band (30–64 Hz) (Alarcao and Fonseca, 2019), and the frequency bands of the bands are divided into slightly different bands by different researchers.

**Table 1:** Bands of EEG signals.

Wave band	Frequency range	Physiological function	Related-emotion	Ref.
δ	1–4 Hz	Unconsciousness, deep sleep	Regulated-emotion	Lapomarda et al. (2022)
θ	4–8 Hz	Deep meditation, consciousness & inspiration	Joyful, anxiety	Sammler et al. (2007) and Tang et al. (2023)
α	8–13 Hz	Relaxation state	Positive, negative, happy, sad	Sammler et al. (2007) and Xu et al. (2024)
β-1	13–22 Hz	Creative thinking, focused states	Positive, negative	Aftanas et al. (2006)
β-2	22–30 Hz	Attention	Happy, sad	Li et al. (2023)
γ	30–64 Hz	Tension state in the brain, high intensity information processing	Happy, sad	Guo et al. (2020) and Li et al. (2023)

Research has shown that the five bands of EEG signals mentioned above are directly or indirectly related to human emotions. While early studies suggested that the δ-band was not connected to people’s emotions, recent research has found that δ wave is closely associated with the emotional state of individuals following emotional regulation and holds promise for use in areas such as music therapy for emotion regulation (Lapomarda et al., 2022).

The acquisition method of EEG signals is mainly categorized into invasive and non-invasive techniques. Invasive measurements require surgical implantation of electrodes to obtain clearer EEG signals, but this method is traumatized to the human body and difficult to widely apply, which is mainly used in clinical medical treatment. Non-invasive is to fit the electrodes to the surface of the head to collect brain signals.

**Figure 2:**
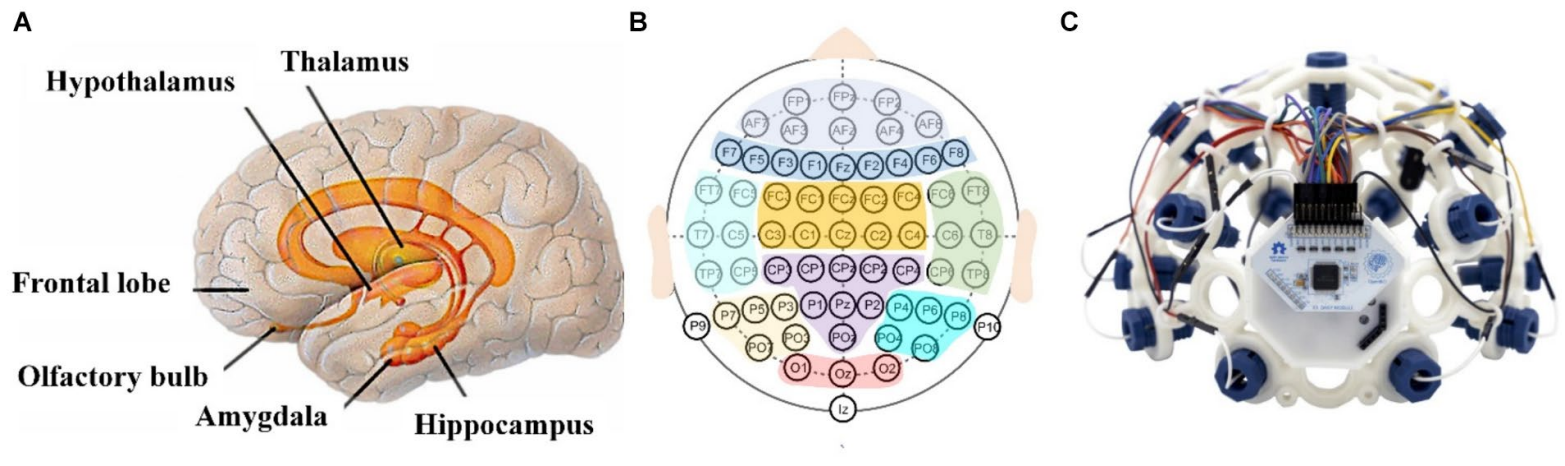
**(A)** The limbic system for emotion, **(B)** the international 10/20 system with nine cortical regions labeled with different colors, **(C)** structure of a typical EEG system from OpenBCI.

Previous research has demonstrated the significance of the limbic system (as in Figure 2A) in regulating human emotions, making it a pivotal area of interest in the field of emotion research (Rolls, 2015). To obtain more comprehensive brain signals, the internationally recognized 10/20 system, shown in Figure 2B, is generally used in the arrangement of electrodes, i.e., the actual distance of adjacent electrodes is 10% or 20% of the distance of the brain skull (Silverman, 1963). In the field of emotion recognition, multi-channel EEG acquisition is commonly utilized, featuring electrode channels ranging from 36 to 64, and a sampling frequency of 500 or 1,000 Hz (Wu et al., 2024). Traditional EEG acquisition system devices are often cumbersome and expensive, which hinders their widespread adoption and use. With the advancement of open source technologies like OpenBCI as in Figure 2C and other EEG acquisition devices, more affordable, user-friendly, and portable options have emerged. Recently, these devices have become increasingly popular in EEG emotion recognition research (Aldridge et al., 2019). To investigate the mechanisms of timely emotional response, both the stimulus source and the acquisition system are typically equipped with time-synchronization devices (Pei et al., 2024).

In music emotion recognition research, non-invasive acquisition schemes are commonly employed. In recent years, wireless wearable non-invasive EEG measurement devices have greatly facilitated EEG-based emotion recognition research (Apicella et al., 2022a; Saganowski et al., 2023). The emergence of these novel EEG acquisition protocols has significant implications for expanding the scope of EEG emotional applications.

### 2.2 EEG signal bands corresponding to different emotions

The brain exhibits diverse EEG response patterns for different emotions, and establishing the relationship between EEG signal bands and various emotional states is a crucial foundation for developing effective classification and recognition models. This correspondence serves as one of the key scientific challenges in the domain of artificial intelligence-based recognition of music-induced emotions. Around 1980, researchers found that EEG’s characteristic signals correlate with human emotional states (Davidson and Fox, 1982), as in Table 1. Subsequently, researchers have investigated the relationship between distinct brainwave frequency bands and diverse emotional states. In 2001, Louis A. Schmidt et al. presented that emotions within valence can be distinguished by evaluating the asymmetry and overall power of α-band (8–13 Hz) from frontal brain EEG signals (Schmidt and Trainor, 2001). In 2007, Daniela Sammler et al. conducted a systematic analysis of the correlation between various EEG frequency bands and emotions. Their findings revealed that θ-band (4–8 Hz) power in the prefrontal lobe is more prominent during happy music stimulation, indicating its significance in emotional processing (Sammler et al., 2007). With the continuous advancement of EEG analysis technology, it has become increasingly apparent that the intricate nature of the brain’s emotional processes makes it challenging to establish precise correlations between different emotions and signals derived from a single brain region or waveform. Certainly, in some specific scenarios, researchers continue to explore and identify the most prominent EEG frequency bands to simplify the challenges associated with emotion recognition.

The application of artificial intelligence in the emotional recognition of music-induced electroencephalography (EEG) holds significant value in two primary aspects. On one hand, the utilization of AI algorithms assists researchers in discerning and selecting the appropriate frequency bands amidst a multitude of options. On the other hand, the deployment of AI algorithms facilitates the exploration of additional effective frequency bands, enhancing the depth and breadth of research in this domain. With the development of artificial intelligence and deep learning technologies, emotion recognition by utilizing various frequency band features from different brain regions has emerged as a prominent and contemporary approach (Pei et al., 2024; Xu et al., 2024). Machine learning based Support Vector Machine (SVM) (Bagherzadeh et al., 2023), Naïve Bayes (NB) (Oktavia et al., 2019), and K Nearest Neighbors (KNN) (Sari et al., 2023) classifier methods have been applied in this field. Deep learning based classification methods such as Convolutional Neural Networks (CNN) (Yang et al., 2019), Recurrent Neural Networks (RNN) (Zhong et al., 2023), Long-Short-Term Memory (LSTM) (Du et al., 2022), and other classification methods have also been used in EEG recognition studies.

## 3 Preprocessing and feature extraction of EEG signals

Extracting effective emotional state information from raw EEG signals is a highly challenging task, given that the signal is a multi-frequency non-stationary signal. EEG preprocessing and feature extraction are essential steps in the recognition algorithms for effective analysis and interpretation of the EEG signals. The purpose of preprocessing EEG signals is to eliminate human motion interference and environmental noise that are unrelated to emotion pattern recognition. This is essential for enhancing the accuracy and robustness of the recognition algorithm.

### 3.1 EEG preprocessing

Noise removal is a crucial objective of EEG signal preprocessing. EEG signals are often vulnerable to interference from various sources such as environmental electromagnetic signals (~50–60 Hz), eye movements (~4 Hz), electrocardiogram signals (ECG, ~1.2 Hz), and so on.

The removal of these noises can significantly enhance the robustness of the EEG model. Usually, these disturbing signals can be filtered out with band-pass filters, wavelet packet filtering, or independent component analysis (ICA) methods as in Table 2. However, researchers have different opinions regarding the signal filtering methods in preprocessing. Some argue that these methods do not eliminate interfering noise, while others believe that these techniques remove noise at the expense of potentially discarding valuable EEG information. To further improve the denoising performance, the artifact subspace reconstruction (ASR) method can be applied to remove the artificial signals. What’s more, the average value of overall electrodes can be applied to subtract from each channel to reduce the system noise (Katsigiannis and Ramzan, 2018). Compared to classical machine learning algorithms, deep learning classification methods for emotion recognition are less influenced by the effects of preprocessing techniques.

**Table 2:** Benefits and drawbacks of EEG preprocessing methods.

Method	Pros	Cons	Ref.
Band-pass filter	Easy to use, fast calculation	Phase delay induced	Er et al. (2021)
Wavelet packet filter	Time-frequency domain information can be analyzed simultaneously	Appropriate wavelet basis functions and scale parameters needed	Balasubramanian et al. (2018)
ICA	Efficient separation of mixed sources, effectively noise remove	Statistical characteristics of the signal source required	Li and Zheng (2023)
ASR	Effectively removes interference from sources other than the scalp surface	High computational complexity, mistake brain signals leading to loss of information	Li et al. (2023)

The most popular open source toolbox for EEG preprocessing is EEGLAB running in the MATLAB environment (Martínez-Saez et al., 2024; Pei et al., 2024; Wu et al., 2024). This interactive toolbox can be applied to process continuous and event-related EEG signals. Moreover, the artifacts from eye movements can be removed with the run independent component analysis (RunICA) algorithm incorporated in EEGLAB based on the independent component analysis (ICA) method (Wu et al., 2024). The expansion of artificial intelligence has led to the integration of EEG signal preprocessing algorithms into a growing array of commercial AI development platforms, including Python, Brainstorm, and Curry.

### 3.2 Time domain feature extraction

Emotional changes in the brain can be influenced by musical stimulation, leading to observable effects on EEG signals. These EEG signals exhibit various time-dependent features, which can be analyzed in the time domain. Time-domain features provide intuitive insights and are relatively easy to obtain. Some categories of time-domain features in EEG analysis include event-related potentials (ERPs), statistical features (such as mean, average, standard deviation, skewness, kurtosis, etc.), rise and fall times, and burst suppression (Stancin et al., 2021; Li et al., 2022b).

**Figure 3:**
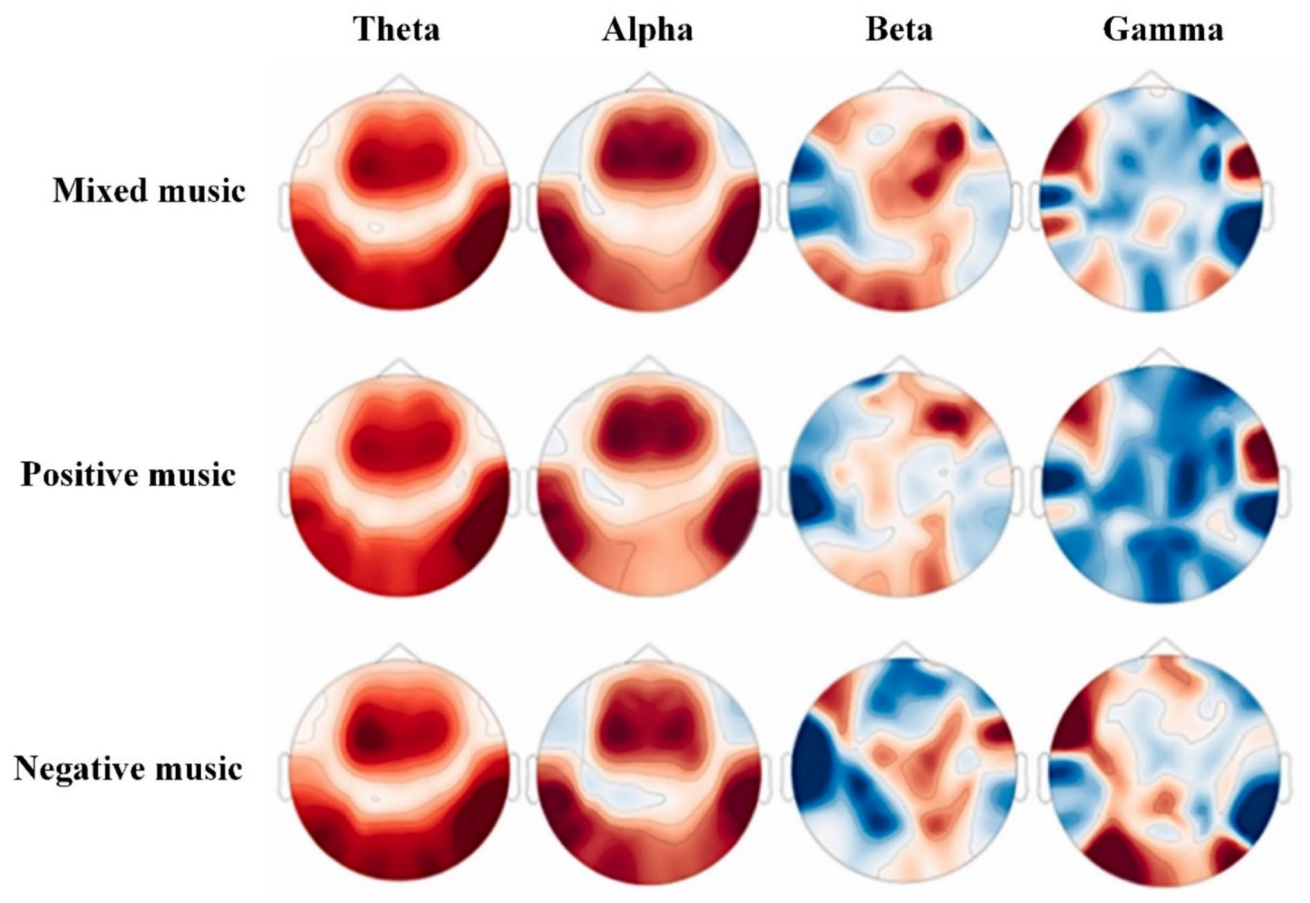
Topographical maps of EEG signals for different types of music (Xu J. et al., 2023).

Time domain features can intuitively capture changes in brain states following music-induced emotions. The active regions corresponding to these emotions can typically be promptly identified through intuitive brain area distribution maps, as in Figure 3, offering valuable insights for the improvement of recognition algorithms. Time domain features are generally preferred in emotion recognition research.

Moreover, event-related potentials (ERPs) are specific patterns of EEG activity that are time-locked to particular sensory, cognitive, or motor events (Martins et al., 2022). They reflect the brain’s response to stimuli and provide valuable information about cognitive processes, which is very helpful in studying the dynamic processes of emotion change with music stimuli. Rise and fall times refer to the duration it takes for the EEG signal to rise from its baseline to its peak (rise time) or fall back to the baseline (fall time). These measures provide insights into the speed of neural activation or deactivation. Currently, there is a relatively limited body of research on the speed, duration, and recovery time of human emotions stimulated by music. It is important to dedicate attention to these aspects in future studies to gain a deeper understanding of the relevant phenomena with the time domain feature of rise and fall times.

By examining these time-domain features of EEG signals, researchers can gain a better understanding of the temporal dynamics of brain activity related to emotional responses to music. The deployment of artificial intelligence algorithms enables the real-time identification of emotions induced by music via EEG signals. Making well-informed choices and applying time-domain features effectively is essential for advancing these studies.

### 3.3 Frequency domain feature extraction

As crucial parameters in EEG emotion recognition algorithms, frequency domain features offer more intricate emotion-related information, including the distribution of energy across different frequency bands. For instance, the energy distribution in high-frequency bands (such as β-band and γ-band waves) tends to increase during pleasurable and exciting emotional states (Li et al., 2018). Analyzing the phase synchronization degree of signals can provide insights into changes in information θ-band wave patterns between brain regions during different emotional states. For example, theta synchronization between the frontal and temporal lobes is associated with pleasant emotions (Ara and Marco Pallarés, 2020). Frequency domain features allow for the analysis of interactions between various brain regions. By calculating correlation features between different brain regions at different frequencies, changes in information exchange patterns between brain regions during different emotional states can be observed (Maffei, 2020). Based on the inter-correlation maps of δ, α and γ-band waves stimulated by six different scenarios, the widest topographical distribution is δ-band, while the narrowest is α-band (Maffei, 2020).

Various techniques are commonly employed for extracting frequency domain features as in Table 3. These include the following methods: Fourier transform, wavelet transform, independent component analysis, and matrix decomposition (Torres et al., 2020; Zhou et al., 2022; Li et al., 2023; Mahmoud et al., 2023). Fourier transform is utilized to convert a time domain signal into a frequency domain signal, providing spectral information such as frequency components and amplitude details (Mahmoud et al., 2023). Frequency domain feature extraction techniques based on the Fourier transform encompass power spectral density (PSD), average power spectral density (APSD), and related features. PSD is usually evaluated within a specific frequency band, considered the most commonly applied feature for classical emotion classifiers (Xu et al., 2024).

**Table 3:** Advantages and disadvantages of frequency domain feature extraction methods.

Method	Pros	Cons	Ref.
Fourier transform	Extract the energy characteristics of different frequency components, effective for spectral analysis of static signals	Unable to capture the time-varying characteristics of the signal, less effective for spectral analysis of non-stationary signals	Wu et al. (2024)
WPD	Provides better local characterization in the time-frequency domain, suitable for the analysis of non-smooth signals, capture transient features of signals	Higher computational complexity, difficult to get appropriate wavelet basis functions and scale parameters	Balasubramanian et al. (2018)
PSD	Intuitive representation of the frequency characteristics.	Higher requirements for source signal quality	Martínez-Saez et al. (2024)

Wavelet transform offers a more versatile and multi-scale approach to signal analysis, delivering both frequency and time information (Bagherzadeh et al., 2023). Frequency domain feature extraction methods associated with wavelet transform involve wavelet packet decomposition (WPD), wavelet packet energy features, and similar characteristics. Independent component analysis serves as a signal decomposition method grounded in independence assumptions, yielding independent frequency domain components post-decomposition (Shu et al., 2018). Frequency domain feature extraction techniques stemming from independent component analysis include frequency band energy distribution, phase synchronization degree, and more. Matrix decomposition is an algebraic signal decomposition method that disentangles the original signal into distinct frequency domain components (Hossain et al., 2023). These techniques enable the extraction of diverse frequency domain features such as spectral characteristics, phase synchronization degrees, correlation features, and so forth. In emotion classification applications, a tailored selection and adjustment of methods and feature combinations can be made based on specific requirements.

The capabilities of artificial intelligence algorithms in mining large-scale data sets not only enable the automatic extraction of frequency characteristics from EEG signals but also reveal the underlying connections between frequency domain signals and emotions.

### 3.4 Time-frequency domain feature extraction

**Figure 4:**
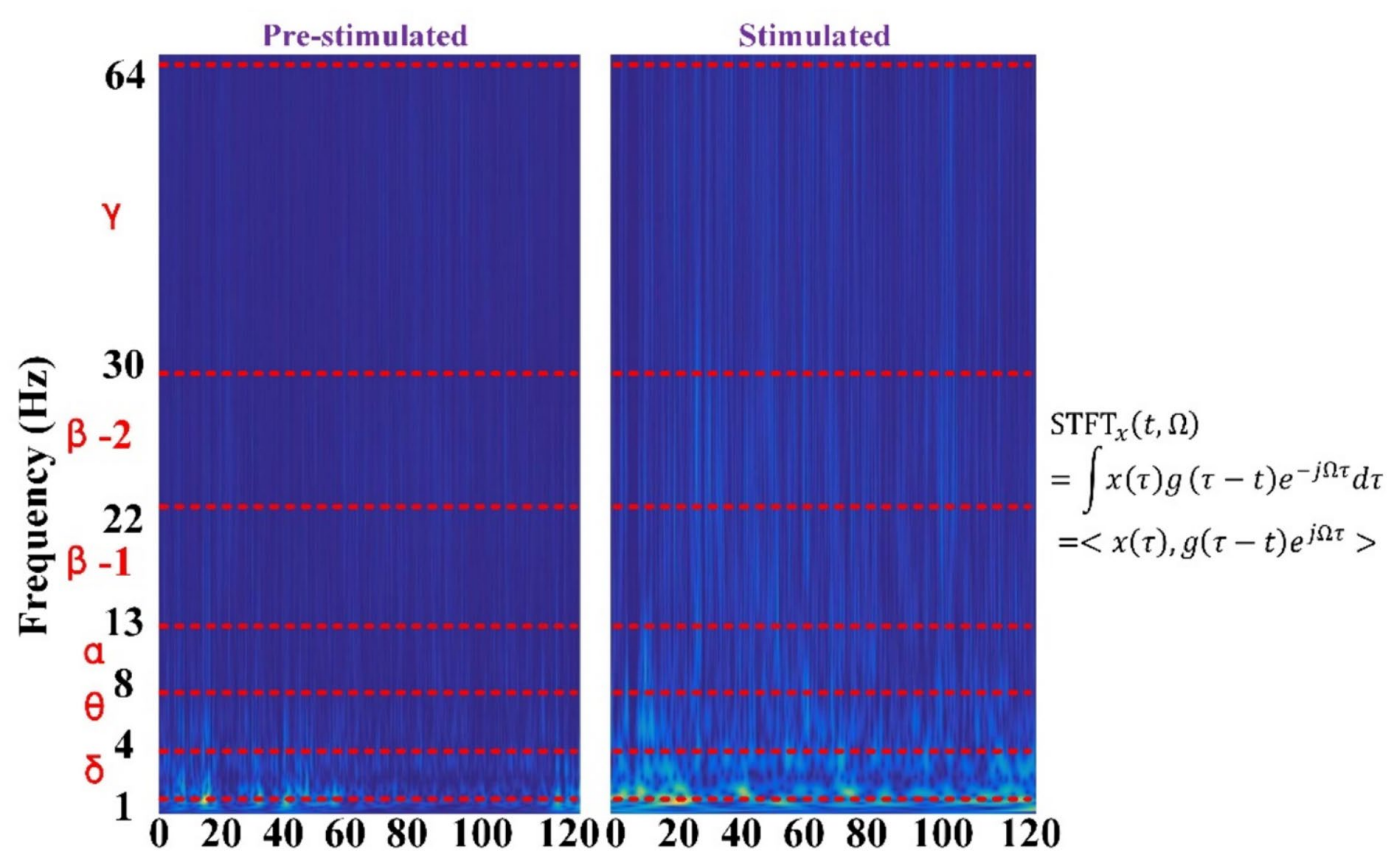
Time-frequency plots before and after stimulation were used by the authors’ research group.

Time-frequency feature extraction methods involve analyzing EEG signal changes in both time and frequency to extract characteristic parameters that capture the dynamic nature of the signal (Bagherzadeh et al., 2023). Common techniques of time-frequency domain features include wavelet transform (Khare and Bajaj, 2021) and short-time Fourier transform (STFT) (Pei et al., 2024). These methods enable the extraction of information across various time scales and frequency ranges, unveiling how signals evolve and frequency as in Figure 4, which also has been applied by our group (Li et al., 2022a).

By extracting time-frequency features, a more comprehensive description of the signal’s dynamic characteristics can be achieved, laying the groundwork for subsequent signal processing and emotion classification analysis.

Time-frequency plots typically encompass a vast array of data points, representing a high-dimensional dataset. The application of artificial intelligence algorithms can automatically discern time-frequency patterns associated with various emotions. This capacity for autonomous learning and data mining enhances the efficacy and reliability of time-frequency plots in the identification of emotions induced by music.

### 3.5 Other advanced features

The development of new emotion-recognition features has been significantly influenced by researchers’ profound insights into the brain’s response to emotions.

Prior physiological and psychological studies have demonstrated that emotions, being intricate mental states, can be discerned by detecting the status of connections between brain regions. In recent years, scholars have advocated for the establishment of a network of brain regions using phase-locked values and the extraction of features from multiple brain functional connectivity networks through the application of the Hilbert transform. These graph features are then fused to facilitate emotion recognition (Li et al., 2019). Based on this concept, researchers have introduced a novel feature called asPLV (averaged sub-frequency phase locking value), which is derived from the Morlet transform method. This feature effectively mitigates the impact of the brain’s inherent frequency oscillations induced by the cognitive processing of emotional fluctuations, thereby enhancing the accuracy of recognizing mood changes induced by music. The calculation process for asPLV is outlined as in Table 4.

**Table 4:** Typical extraction process flow of asPLV.

**Input:**	
	Electroencephalogram (EEG) data on *N* subjects. *C* = {*C_1_, C_2_, …, C_N_*} ∈ *R^N^_S_^×N^_C_^×N^_E_*: A set of EEG data C with sample points *N_S_*, channels *N_C_* and epochs *N_E_*.
**Procedures:**	
	**For** *j* = *1, 2, …, N_E_* do
	*c_j_* ∈ *R^N^_S_^×N^_C_* → a single epoch *c_j_* from *C*
	*d_j_* = {csd(*c_j_*)}, *d_j_* ∈ *R^N^_S_^×N^_C_* → After preprocessing, EEG signals are calculated using CSD filter csd (i.e., the surface Laplace transform). *d_j_* is the filtered signal.
	*x_j_* =, *x_j_* ∈ *R^n × N^_C_^×N^_f_^×N^_S_* → The signal of each frequency band after time–frequency decomposition is calculated by wavelet time–frequency decomposition wavelet, where *N_f_* is the number of decomposed frequencies, and *n* is the number of frequency segments (i.e., theta, alpha, beta, gamma, etc.).
	*p_j_* = {*asPLV*()}, *p_j_* ∈ *R^n × N^_C_^×N^_f_^×N^_S_* → asPLV calculation, where *p_j_* is asPLV feature.
	**End for**
**Output:**	
	P = {*p_1_, p_2_, …, p_N_*} ∈ *R^n × N^_C_^×N^_f_^×N^_S_* → asPLV feature for each frequency band converts from set *C*.

In recent years, scholars have discovered that the spatiotemporal characteristics of EEG play a crucial role in emotion recognition. Many studies have introduced novel spatiotemporal features based on self-attention mechanisms (Zhou and Lian, 2023). As our comprehension of the neural mechanisms underlying emotional responses deepens, these new features are critical for enhancing the accuracy of emotion recognition.

Other than these commonly applied features already discussed, artificial intelligence algorithms excel in processing multidimensional data, enabling the discovery of innovative feature metrics. These algorithms hold great promise in identifying individual-specific traits, and crafting features that are sensitive to the distinctive attributes of each individual.

## 4 Emotion data source and modeling

Auto-emotion recognition can be realized by integrating various data sources and emotion models. This is important for the development of music-induced emotion recognition and its application areas. In the realm of music-induced emotion recognition, emotional data sources form the foundation for acquiring emotion related in sights, while models serve as the essential tools for processing and analyzing this valuable information (Saganowski et al., 2023; Xu J. et al., 2023).

### 4.1 Data sources for music-evoked emotion classification

In recent years, in order to promote research on music-induced emotions, a series of databases of music-triggered emotions have been established, with emotion labels provided by psychologists. Although these databases can be used for music-triggered emotion research, they lack a unified criterion. Based on the EEG method of emotion discrimination, researchers also have established emotion databases containing EEG signals. These open source databases are not only important resources for conducting research on music-triggered emotions, but can also be used to evaluate the performance of different EEG algorithms. Table 5 shows some common open source music emotion databases and their characteristics.

**Table 5:** Open-source music emotion databases for music-induced emotion classification.

Database name	Source	Number of subjects	Features of the songs	Emotional classification methods
AMG1608^1^	Chen et al. (2015)	665	1,608 music clips	VA coordinate dimension modeling
CAL500^2^	Turnbull et al. (2008)	>1,500	502 songs, western pop	Key emotional vocabulary
DEAM^3^	Aljanaki et al. (2017)	21 Teams	1802 songs, over 12 categories	Keywords combined with statistical methods
emoMusic^4^	Soleymani et al. (2013)	>10,000	1,000 songs, 8 categories	Time-varying continuous VA coordinate dimension models
Emotify^5^	Zentner et al. (2008)	>4,000	400 songs, 4 categories	Rating labeling of Likert scales
DEAP^6^	Koelstra et al. (2012)	32	40 pieces of music	VA models of physiological signal binding
IADS^7^	Yang et al. (2018)	207	935 pieces of digital music	Manual multiple mood scale

1 http://amg1608.blogspot.com/.

2 https://paperswithcode.com/dataset/.

3 https://cvml.unige.ch/databases/DEAM/.

4 http://www.multimediaeval.org/.

5 http://www2.projects.science.uu.nl/memotion/emotifydata/.

6 http://www.eecs.qmul.ac.uk/mmv/datasets/deap/.

7 https://sites.google.com/view/iads-e/.

**The AMG1608 database** is a database containing acoustic features extracted from 1,608 music clips of 30s as well as emotion annotations provided by 665 subjects, consisting of 345 females and 320 males. The database used a dimensional emotion model with validity and arousal (VA) as the coordinates in the emotion annotation, and the subjects annotated the emotional state of each music clip. The dataset contains two subsets of emotion annotations from National Taiwan University and Amazon Turkish Robotics and is characterized by a large amount of data capable of being publicly accessible and can be used for music emotion recognition research.

**The CAL500 database** is composed of 500 Western songs’ clips written by 500 different artists. For the emotion annotation of the music, 174 music-related semantic keywords were used, and at least three subjects annotated keywords for each song. These annotated words were also post-processed algorithmically to constitute a vector of annotated words and weights, ensuring the reliability of the annotation labels. The dataset is able to satisfy the fine granularity and differentiation required in music emotion recognition research.

**The DEAM database**, which labels musical emotions in terms of valence and arousal (VA) coordinates, has 1,802 songs licensed under the Creative Commons (CC) license. This music library contains categories such as rock, pop, soul, blues, electronic, classical, hip-hop, international, experimental, ethnic, jazz, country, and pop. The emotion annotations for these songs were made by 21 active teams from all over the world, and these annotations were statistically processed to form a database that can be used for music emotion research.

**The emoMusic database** contains 1,000 audio tracks in MP3 format licensed under the Creative Commons (CC) License in eight different genres: blues, electronica, rock, classical, folk, jazz, country, and pop, with 125 tracks in each genre. The emotion labeling of the music was evaluated using valence and arousal (VA) model, where valence indicates positive and negative emotions and arousal indicates emotional intensity. The database collects time-varying (per second) continuous VA rating data, with each song containing at least 10 thematic annotations. The database can be utilized for the conduct of research related to music emotion annotation and other related studies.

**The Emotify database** contains 100 pieces of music from each of the four genres of classical, rock, pop, and electronic music randomly selected from a collection of music containing 241 different albums by 140 performers. The database used the Geneva Emotional Music Scale (GEMS), in which subjects labeled the emotions of the music using a Likert scale using a scale of 1–5. The database provides case studies and information on the effects of other factors on evoked emotions (gender, mood, music preference).

**The DEAP database** is a music emotion recognition database based on an EEG emotion recognition method, which was built together by a consortium of four universities from the UK, the Netherlands, and Sweden, and records EEG and physiological information from 32 subjects who watched a series of forty 1-min music video clips. The database was selected as a semi-automatic stimulus selection method based on emotional labeling is open access to academics and can facilitate research related to emotional stimulation in modern music.

**The IADS database** is the International Emotionally Digitized Sound Database, which is divided into two distinct phases. The initial Phase I database, established in 1999, contains a modest collection of data that has seen limited use in contemporary times. In contrast, Phase II is an expansive compilation of 167 digitally captured ambient sounds that are frequently encountered in everyday life, such as the joyful laughter of an infant, the rhythmic sounds of cooking, and the dramatic rumble of thunderstorms, with each sound clip precisely lasting 6 s. The collection is meticulously annotated, with each piece of digital audio being evaluated by participants through a self-assessment approach that utilizes the affective dimensions of the Valence-Arousal (VA) model.

At present, these databases of music emotions are mainly based on foreign music libraries, the situation is related to the importance of music in the relevant regions, and the establishment of music databases based on Chinese musical compositions is yet to be carried out. Due to the complexity of the signal measurement and classification of EEG in the early stage, there are fewer studies for EEG music-induced emotion recognition. Enabled by artificial intelligence, EEG-based music emotion recognition can help to expand the establishment of databases as soon as possible, and it can help more researchers to apply the established databases. Currently, besides the above common music-induced emotion databases, many researchers also use their own designed libraries to carry out personalized research in the study of music-induced emotions with EEG. As artificial intelligence technology advances, it facilitates the integration of EEG data with other modalities of data, thereby enriching the dimensions of information within the database. The application of data augmentation techniques helps to enhance the generalization capability of models built from the database. With the progression of research in EEG-induced emotion recognition, artificial intelligence can also assist in the automatic consolidation and updating of databases, providing technical support for the construction of more comprehensive, accurate, and holistic datasets.

### 4.2 Emotion classification models

**Figure 5:**
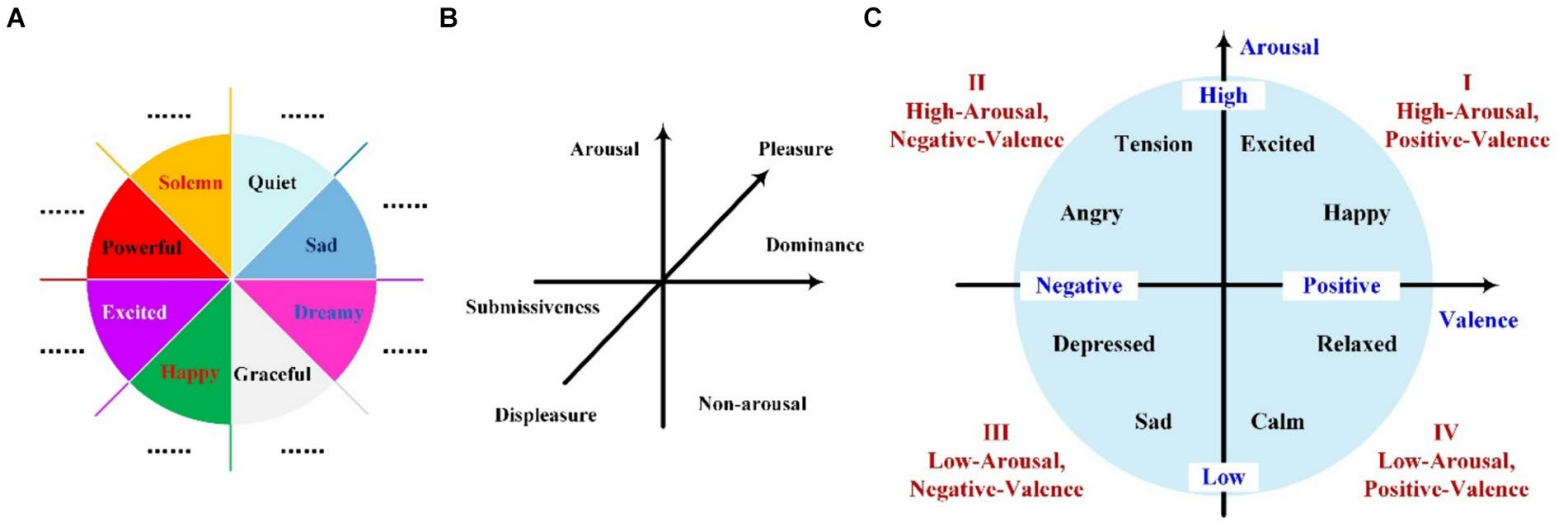
Schematic diagrams of common emotion recognition models. **(A)** Hevner’s model, **(B)** PAD model, **(C)** Thayer’s model.

To address the challenge of quantifying the emotions elicited by music, researchers have developed a variety of models specifically designed to capture the nuances of music-induced emotions. These models aim to provide a structured approach to understanding and measuring the complex emotional responses that music can evoke. The classical music emotion models for analysis are shown in Figure 5, including the Hevner model (Hevner, 1936), the Pleasure Arousal Dominance (PAD) model (Russell, 1980), and the Thayer model (Thayer and McNally, 1992). With the development of artificial intelligence technology, some algorithm-based emotion classification approaches also have been proposed.

In computerized categorization studies of musical emotions, the first psychological Hevner emotion classification model was proposed in 1936 (Hevner, 1936). This model classifies music emotion states into eight categories: Solemn, Sad, Dreamy, Quiet, Graceful, Happy, Excited, and Powerful as in Figure 5A. Each category can be further subdivided into more detailed and extensive emotion words, with a total of 67. This emotion classification model was set up considering musicology and psychology and is more abundant in the selection of emotion keywords, which is helpful for the research of emotion recognition in musical works. Hevner is a discrete emotion classification model that is often used as an emotion label for songs in music-induced emotion recognition research. However, due to the large number of labeling categories of the model and the relatively low variability of physiological properties of some categories, this model is seldom applied in EEG-based music emotion recognition studies, but it can be considered in relevant studies for featured music.

Among the classification models of musical emotions, PAD is a three-dimensional measurement model that was proposed in 1980. As in Figure 5B, this model establishes three main dimensions of Pleasure-Displeasure, Arousal-Nonarousal, and Dominance-Submissiveness, which indicate the direction of the emotion, the degree of neurophysiological activation, and the degree of individual feeling, respectively. This categorization provides a continuous quantitative evaluation method, which has been widely used in psychological and emotional brand evaluation (Yang et al., 2020) but has not been used much in the actual evaluation of musical emotions. Thayer’s model is a two-dimensional model that suggests different emotions are classified based on two-dimensional underlying dimensions, i.e., Energy awakening and Tension awakening. Using stress as the horizontal coordinate and energy as the vertical coordinate, emotions are categorized into four zones: vitality, anxiety, contentment, and depression as in Figure 5C. This model is proposed based on a psychological perspective and describes the music mood according to quantitative thinking, which is often used to classify the mood of audiophile music in MP3 and WAV formats (Brotzer et al., 2019). Since this model has fewer classification dimensions compared with other mentioned models and is more visible on the emotional response, they are well suitable to be used for EEG recognition of music-induced emotions. Besides the above classical emotion classification models, some researchers have also used probability distribution (Kim et al., 2022), neural network method (Yang, 2021), linear regression (Griffiths et al., 2021), and inverse word pairs (Liu et al., 2019) approaches to characterize the emotions of music. The probability distribution method describes the emotions corresponding to the song in the emotion description space, which gives a more comprehensive and intuitive description of the song. The ranking is utilized to order the emotion descriptions of songs according to the degree of relevance of the emotions expressed, which is convenient to provide a quick description method. The antonym pairs can give a relatively objective description of the mood of the music. Several researchers have currently extended discrete and multidimensional models for music emotion description based on these ideas.

These new classification models are related to the development of emotion categorization algorithms and have a large potential for application in the field of EEG music-induced emotions. Different emotion models can be used to describe the classification of emotions in different states, meanwhile, there are some intersections between these different models. For different practical applications, people need to choose the appropriate emotion classification models according to the research scenarios and artificial intelligence algorithms.

### 4.3 Emotional intensity model

**Figure 6:**
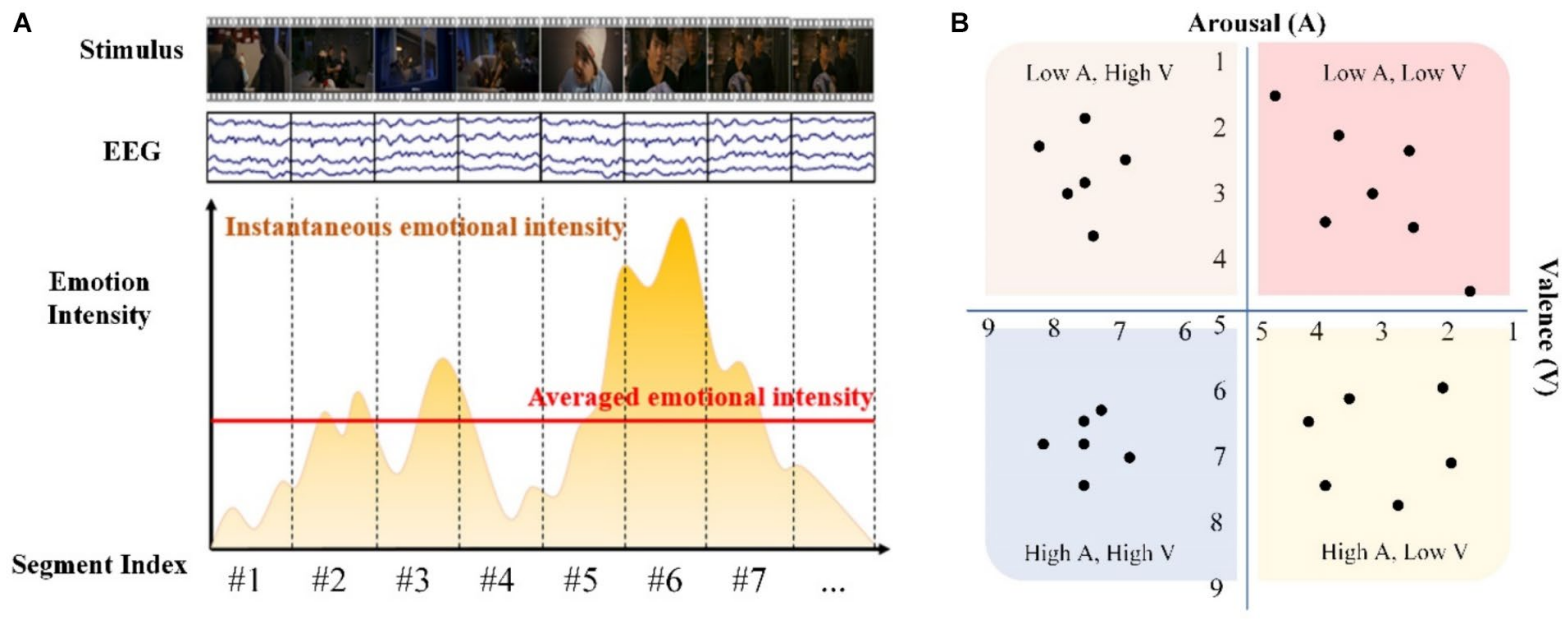
**(A)** The instantaneous emotional intensity of stimulated emotion dynamic process (Gan et al., 2024), **(B)** schematic of a fine-grained emotional division.

Emotional intensity models were applied to quantitatively delineate the depth of emotions experienced by individuals in specific circumstances, forming the cornerstone for emotion recognition and prediction models. The discourse on quantifying emotional intensity emerged around 1990, advocating for a shift from solely focusing on subjective emotional aspects to incorporating physiological and observable behaviors as metrics of intensity (Russell, 1980; Sonnemans and Frijda, 1994). In 1994, Frijida introduced a five-dimensional model for scrutinizing subjective emotions, encompassing dimensions such as the frequency and intensity of re-collected and re-experienced emotions, latency and duration of emotions, intensity of actions and propensities, as well as actual behaviors, beliefs, and behavioral changes (Sonnemans and Frijda, 1994). In recent years, researchers have explored the use of emotional intensity modeling to study the instantaneous dynamic processes in the brain under external stimuli, as in Figure 6A (Gan et al., 2024). This innovative approach provides a new approach to the study of the neural mechanisms and processes of music-induced emotions.

Despite offering a theoretical framework for objectively describing emotional states, the model’s impact was limited due to the scarcity of physiological emotion measures at that time.

A prevalent theoretical framework in recent years for elucidating emotional intensity is Russell, 1980 proposition that emotional experiences can be depicted in a two-dimensional space defined by emotional valence (positive vs. negative emotions) and arousal levels (high vs. low) (Russell, 1980). Building upon this framework, researchers have delved into refining each dimension to achieve a nuanced portrayal of emotions illustrated in Figure 6B, laying the groundwork for leveraging artificial intelligence in digitally characterizing emotions (Reisenzein, 1994; Zhang et al., 2023). Physiological emotional intensity indices such as EEG, ECG, and MRI are not only valuable for emotion recognition but also serve as essential tools for studying the dynamic processes and physiological mechanisms underlying music-induced emotional changes (Ueno and Shimada, 2023; Wu et al., 2024).

## 5 Artificial intelligence algorithms for EEG emotion recognition

Music-induced EEG-based emotion classification research can be considered an artificial intelligence classification task, where the selection of appropriate classification algorithms is a crucial element in the current research on EEG-based emotion classification. These algorithms are not only the topicality of emotion classification research in EEG but also serve as an important foundation for further research into music-induced emotions (Khabiri et al., 2023).

### 5.1 Classical machine learning algorithms

Based on the EEG feature signals of music-induced emotions, various classical machine learning classification methods have been commonly used to achieve relatively good classification accuracy. These methods include classical classifiers such as Bayes classifier (BC) (Koelstra et al., 2012), linear regression (LR) (Zheng and Lu, 2015), support vector machines (SVM) (Bagherzadeh et al., 2023), K Nearest Neighbor (KNN) (Khabiri et al., 2023), and random forests (RF) (Pei et al., 2024), as in Table 6.

**Table 6:** Several classical machine learning methods applied in music-induced emotion classification.

Classifier	Characteristics	Ref.
BC	Effective with small sample data, high requirements for filtering features, small number of classifications, difficult to handle multi-featured music emotion multi-classification tasks	Koelstra et al. (2012)
LR	Simple and strong interpretability, weak fitting for nonlinear models and sensitive to abnormal values	Zheng and Lu (2015)
KNN	Non-parametric machine learning algorithms, easy to implement, high computational complexity, sensitive to outliers, low accuracy	Khabiri et al. (2023)
RF	Multi-dimensional data, high accuracy, complex and difficult to interpret	Pei et al. (2024)
SVM	Excellent performance for classification problems offering good accuracy and generalization, easy to interpret and effectively handles high-quality features, difficult to optimize parameters, sensitive to data distribution.	Bagherzadeh et al. (2023)

These algorithms have been extensively employed in the field of emotion classification research and have shown promising results in accurately classifying mu-sic-induced emotions (Dadebayev et al., 2022). One of the commonly used supervised classification algorithms for music sentiment is the K Nearest Neighbor (KNN) algorithm. KNN, as a supervised learning algorithm, is highly versatile and easy to understand. It is robust to outliers and has a simple principle. However, the computational results of the KNN algorithm can be influenced by the training set samples as well as the value of K, which represents the number of nearest neighbors considered for classification. It is important to carefully select the appropriate value of K and ensure the representativeness and quality of the training set to achieve accurate classification results in music sentiment analysis. Another commonly used classical classifier for music sentiment analysis is the Support Vector Machine (SVM). When using SVM for classification, the choice of the kernel function has a significant impact on its performance. By mapping the features nonlinearly to a high-dimensional space using the kernel function, SVM improves the robustness of the music emotion recognition algorithm (Cai et al., 2022). SVM is particularly effective in handling high-dimensional data, making it suitable for achieving better classification results in music EEG emotion recognition compared to KNN.

Classical machine learning algorithms exhibit strong interpretability, high data efficiency, and low computational resource requirements in music emotion recognition research. These characteristics are highly desirable for studying the neural mechanisms of music-induced mood changes. However, in practical applications of music emotions, these models often suffer from poor generalization performance and require improved accuracy.

### 5.2 Deep learning algorithms

Although machine learning algorithms have been used for emotion recognition and have shown improvements, there are still challenges such as feature extraction, stability, and accuracy. However, the emergence of deep learning methods in recent years has provided a promising approach for EEG-based music emotion recognition research. Deep learning algorithms, characterized by their strong learning ability, have shown great potential in this field.

One notable deep learning algorithm applied in EEG-based music emotion recognition research is Convolutional Neural Networks (CNN). CNN extends the network structure of artificial neural networks by incorporating convolutional layers and pooling layers between the input layer and the fully connected layer. This architecture allows CNN to automatically learn and extract relevant features from the input data, making it suitable for analyzing complex patterns in EEG signals. By leveraging deep learning techniques, researchers can enhance the performance of music emotion recognition systems. Deep learning algorithms can effectively handle the high-dimensional and time-varying nature of EEG signals, leading to improved accuracy and stability in emotion recognition tasks. Moreover, with the ability to capture hierarchical representations, CNN can capture both local and global features in EEG data, enabling a more comprehensive analysis of music-induced emotions (Moctezuma et al., 2022; Mahmoud et al., 2023). With the development of deep learning algorithms, a variety of deep learning models have been developed and applied to EEG-based music-induced emotion recognition, including recurrent neural networks (RNN) (Dar et al., 2022), long and short-term memory networks (LSTM) (Du et al., 2022), gated recurrent neural networks (GRNN) (Weerakody et al., 2021) and autoencoder (AE) (Liu et al., 2020). The properties and applications of these reported deep learning algorithms are summarized in the following Table 7.

**Table 7:** Properties of typical deep learning methods applied in music-induced emotion classification.

Algorithm	Properties and applications	Ref.
CNN	Extract key temporal and spatial features, fail to extract sufficient temporal dynamic information	Mahmoud et al. (2023)
RNN	Captures temporal correlation of signals, complex training process, prone to gradient loss, difficult to deal with long-term dependencies	Dar et al. (2022)
LSTM	A gating control mechanism included, captures long-term dependencies, many parameters needed and long training time	Du et al. (2022)
GRNN	Convenient to deal with time-series data, effective in dealing with long-term dependencies, highly interpretable, high training complexity, difficult to adjust parameters, high requirements on data quality	Weerakody et al. (2021)
AE	Deal with complex features, less requirements of EEG dataset, easy to overfitting, difficult to tune parameters	Liu et al. (2020)

The deep learning algorithms employed in EEG recognition of music-induced emotions demonstrate excellent generalization capabilities and data insensitivity, essential for the practical application of such emotions. While deep learning algorithms typically lack interpretability, recent advancements like GRNN (Weerakody et al., 2021) and RNN (Dar et al., 2022) can effectively capture the temporal aspects of EEG data, offering a novel approach to investigating the transient characteristics of music-induced emotions.

### 5.3 Model optimization and fusion strategies

Previous studies have demonstrated that classical machine learning algorithms as well as deep learning algorithms each possess their unique strengths and weaknesses in EEG-based music-induced emotion recognition research. To address the research and application requirements in related domains, researchers have investigated fusion strategies involving diverse algorithms.

To enhance the precision of emotion recognition, researchers have delved into a hybrid deep learning framework combining gated recurrent unit (GRU) and CNN to leverage the strengths of both methodologies. The conventional GRU model is excellent in handling time series data, while the CNN model is adept at capturing spatial features within the data. During the implementation phase, researchers opted to retain all feature information outputted by the GRU and extract spatial information from the temporal features using the CNN model. Ultimately, they achieved a recognition average accuracy of 87.04% (Xu G. et al., 2023). Based on the brain’s functional network of emotional activity, researchers proposed a multi-feature fusion method combining energy activation, spatial distribution, and brain functional connectivity network features. In the study, the SVM model-based fusion of power activation features of differential entropy (DE), spatial features of common spatial patterns (CSP), five frequency features, and phase synchronization features of EEG phase-locked values (PLV) achieved classification results with an average accuracy around 85% (Pan et al., 2022).

**Figure 7:**
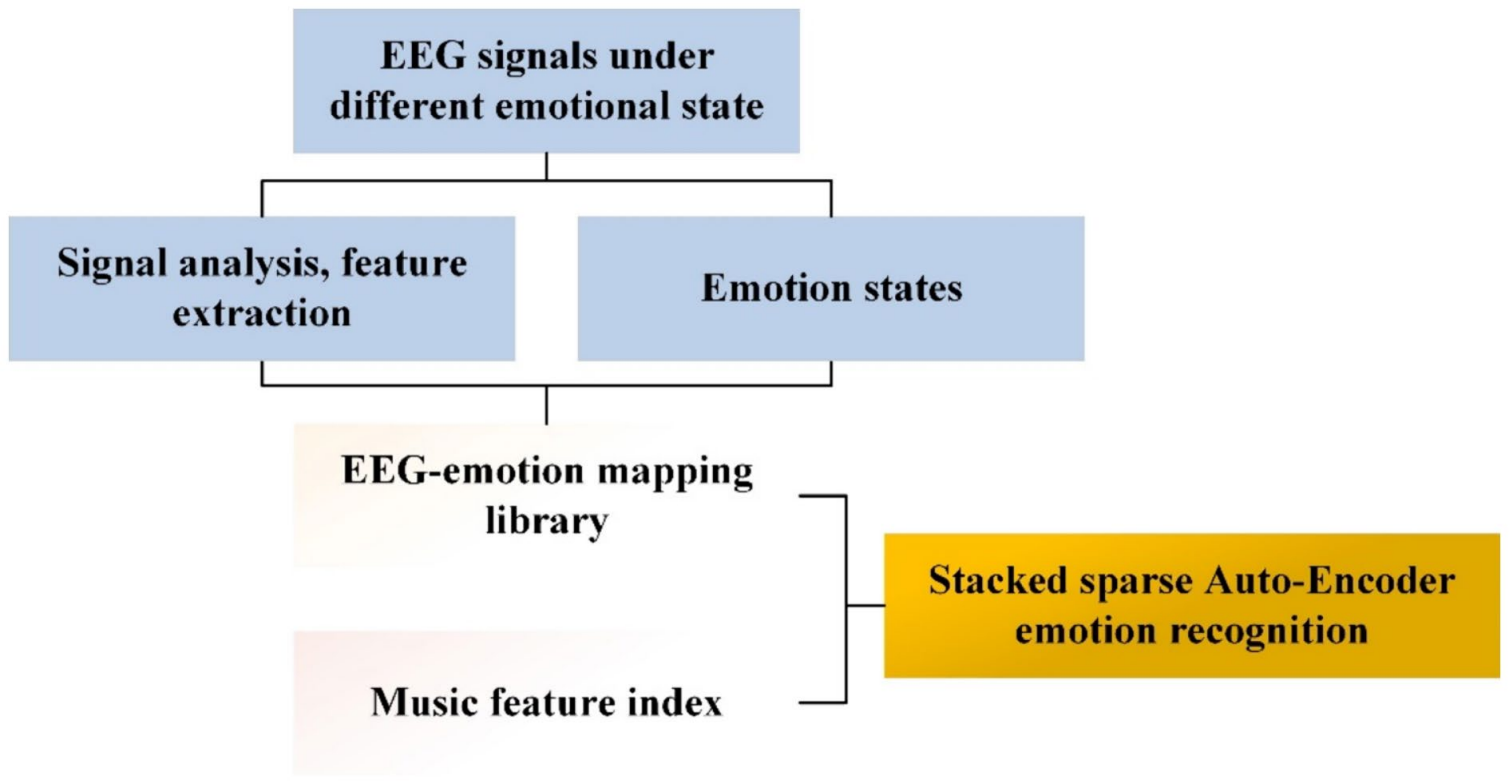
EEG musical staff model process flow chart.

To realize the fusion between different machine learning algorithms, it can be achieved by combining multiple basic classifiers for better performance, fusing different algorithmic training models for model fusion of multiple ones, and also by joint training of multiple neural network models for fusion of different algorithms, etc. In addition to these fusion approaches mentioned above, some researchers have also considered about the optimization method from a music-induced emotions perspective. A pentameter-based EEG music model was proposed. The model constructs a multi-channel EEG sensor network and records the EEG of individuals in various emotional states to establish a mapping library of EEG and emotions. Subsequently, the music pentameter model is employed to adaptively segment the time-domain EEG signal, transforming the EEG signal. The time-frequency features of the EEG signal, such as amplitude, contour, and signal frequency, are quantitatively represented in a normalized musical space (Li and Zheng, 2023). The arithmetic modeling process is described as in Figure 7.

### 5.4 Algorithm comparison and evaluation

Evaluating these various algorithms used for music-induced emotion EEG is very difficult. There is no clear consensus on an optimal algorithm, and several metrics can be employed to evaluate the selected algorithmic model to satisfy the requirements for various applications. The accuracy of emotion recognition is a fundamental evaluation metric, reflecting the model’s performance in correctly predicting samples compared to the total number of samples and overall classifying emotions. Precision and recall, as evaluation metrics for binary classification problems, aid in assessing the model’s performance across different categories. With the expansion of applications and the development of algorithms, developing new criteria is also an important part of future research.

## 6 Application examples and analysis

EEG-based music emotion recognition has emerged as a multidisciplinary research area at the intersection of medicine, psychology, and computer science. This field explores the use of EEG signals to detect and classify the emotional responses evoked by music. The insights gained from EEG-based music emotion recognition have profound implications across various domains.

### 6.1 Music therapy

Music therapy is a therapeutic approach that harnesses the power of music and musical activities to promote both physical and mental well-being. The use of music for healing purposes has a long history, dating back to ancient civilizations like Greece, Rome, Egypt, and China. In more recent times, the formal recognition of music as a legitimate form of therapy began with the establishment of the American Music Therapy Association in 1944 (Taylor, 1981). This marked a significant milestone in acknowledging music therapy as a valid and effective treatment modality within modern society. To broaden the scope of music therapy, the American Music Therapy Association took a significant step in 2005 by introducing the Research Strategic Priority (RSP) program. The primary objective of this initiative is to delve into the physiological evidence supporting the effectiveness of music therapy in both practical and theoretical contexts. In 2013, a team of researchers from Finland conducted a study to investigate the impact of music on the activity of frontotemporal areas during resting state in individuals with depression. The study utilized EEG-based recognition of music-induced emotions as a methodological approach (Fachner et al., 2013). In 2018, a team of Spanish researchers conducted a study to evaluate the emotional response to music in patients with advanced cancer using EEG signals. The study aimed to demonstrate the positive emotional impact of music therapy on these patients (Ramirez et al., 2018). In 2020, a team of Canadian researchers conducted a study to explore the potential of music-induced emotions in alleviating psycho-cognitive symptoms of Alzheimer’s disease. The study involved combining EEG analysis methods to investigate how music activates the brain system, reduces negative emotions, and increases positive emotions. By analyzing EEG signals, the researchers were able to assess the emotional states induced by music. They found that music had a significant impact on the participants’ emotional well-being, with the potential to reduce negative emotions and increase positive emotions (Byrns et al., 2020).

### 6.2 Neuroscience

Brain science is a rapidly growing field of research that offers valuable insights into human thinking, behavior, and consciousness. One area of study within brain science is the investigation of how music stimulates the brain, which has been recognized as a notable example of this research. In 1992, French and German scientists conducted a groundbreaking EEG analysis study to examine the effects of music stimulation on the brain. The study revealed a fascinating phenomenon: different types of music had varying impacts on the intensity of EEG signals across different frequency bands (Steinberg et al., 1992). In 2016, a team of Indian researchers conducted a study using EEG to investigate the effects of Hindustani music on brain activity in a relaxed state. The results of the study revealed that Hindustani music had a significant effect on the listeners’ arousal levels in all activities stimulated by the music. The EEG analysis indicated an increase in brain activity in response to the music, suggesting that it had a stimulating effect on the listeners (Banerjee et al., 2016). In 2019, a group of Indian scholars delved into research on the reverse inversion of brain sounds by utilizing Hindustani classical music. They recognized the profound emotional impact of this music and sought to explore the correlation between EEG signals and musical stimuli. By leveraging the real-time recording capability of EEG, researchers from the fields of psychology and neurology conducted studies to analyze the neural mechanisms underlying the stimulation of music, both in positive and negative contexts. These investigations have significantly contributed to the advancement of brain science research (Sanyal et al., 2019).

In the early stages, EEG, as a direct signal of brain activity, was employed by neuroscientists to conduct exploratory studies on functional brain regions associated with impaired musical ability caused by brain dysfunction. This utilization of EEG monitoring technology has played a pivotal role in advancing our understanding of the brain’s mechanisms involved in music processing (Vuust et al., 2022). These initial findings laid the technical groundwork for subsequent research on EEG-based music emotion recognition. With a focus on music-induced emotions, researchers have endeavored to further investigate the realm of music-induced emotions using EEG technology (Gomez-Canon et al., 2021). From the perspective of music therapy, the utilization of EEG signals offers direct evidence regarding the process of music-induced emotions. By analyzing EEG signals from various brain regions corresponding to different emotions, researchers can obtain more detailed physiological information that aids in the interpretation of the brain mechanisms involved in music therapy. This application of EEG signals provides valuable insights into understanding the effects of music on emotional states and enhances our knowledge of the therapeutic potential of music (Byrns et al., 2020; Fedotchev et al., 2022).

### 6.3 Others

Emotions play a crucial role in human experiences, behaviors, health, and social interactions. Music, a language of the human mind, has the power to vividly and imaginatively express various emotions such as happiness, sadness, and more, and can greatly influence listeners’ emotional state. In recent years, there has been significant progress in understanding music-induced emotions and their psychological and neurological mechanisms.

In clinical medicine, this research can contribute to the development of personalized music therapy interventions for mental health disorders, neurorehabilitation, and stress management. It can also aid in diagnosing and monitoring emotional disorders such as depression and anxiety. In the realm of brain science, EEG-based music emotion recognition provides valuable insights into the neural mechanisms underlying emotional processing and music perception. These findings can enhance our understanding of how the brain responds to music and its impact on emotional well-being. Moreover, in the field of music information, this research can improve music recommendation systems, enhance user experiences, and facilitate music healing approaches. By tailoring music selections based on an individual’s emotional responses, music platforms can offer personalized and therapeutic listening experiences. Overall, EEG-based music emotion recognition holds immense potential for diverse applications in fields like clinical medicine, brain science, and music information. It represents a promising avenue for advancing our understanding of the complex relationship between music and emotions and harnessing music’s therapeutic benefits.

Furthermore, for some long-term music healing processes, the real-time sensitivity of EEG to emotional signals induced by music stimulation can provide evidence for the effectiveness of certain therapeutic methods. This evidence can facilitate the development, correction, and smooth dissemination of related therapeutic techniques. By monitoring changes in EEG signals throughout the music therapy process, researchers can evaluate the effectiveness of different therapeutic methods and fine-tune them accordingly. This approach enhances the precision and efficacy of music therapy, allowing for optimized treatment plans that cater to individual needs (Byrns et al., 2020). For music researchers, the individual variability in EEG emotion detection allows for personalized categorization and annotation of musical emotions. This capability is crucial not only for music composition and information retrieval but also for guiding the development of more immersive multimedia environments. By leveraging EEG data to understand how individuals uniquely experience and respond to musical emotions, researchers can enhance the creation of tailored musical experiences and enrich the design of multimedia environments that resonate with diverse emotional responses (Yu et al., 2022).

## 7 Discussion and conclusions

Based on the mentioned model, researchers were able to carry out systematic research on the study of the emotional impacts of the same music on different listeners, the study of the emotional impact of various types of music on the same listener, and the key parameters of music-stimulated emotions. Previous researchers have conducted various studies in terms of music-induced emotion classification models, music-induced datasets, training and classification of emotion models, and so on.

As an interdisciplinary challenge, research on EEG-based music-induced emotion recognition has emerged as a valuable approach for real-time and effective assessment of emotional responses to music. This innovative research not only offers new technical tools for studying music-related emotions but also provides a controllable research paradigm applicable to brain science and other fields. In recent years, researchers from various disciplines have made significant progress in addressing this complex issue. By approaching the problem from different perspectives, they have achieved notable results. However, during these investigations, several limitations have also been identified.

Compared to other signals commonly used for music emotion recognition, such as audio signals, facial expressions, heart rate, and respiration, EEG signals have distinct advantages. EEG signals belong to physiological signals of the central nervous system, which are typically not under conscious control. Consequently, they can provide information about the current emotional state of an individual that cannot be deliberately concealed. Furthermore, EEG signals offer several benefits when compared to other methods of detecting physiological signals of the central nervous system. EEG is a relatively mature technology that has been extensively studied and validated. It is also portable, non-invasive, and cost-effective, making it practical for use in various research and real-world settings.

As EEG monitoring hardware and recognition algorithm software technology continue to evolve, the advantages of personalization, real-time analysis, and the convenience of using EEG to recognize music-induced emotions will be further explored in various application fields. The growing sophistication of EEG technology opens up new possibilities for research and practical implementation of music-based therapies, multimedia environments, and personalized music experiences. As such, the continued development and refinement of EEG-based music emotion recognition has the potential to revolutionize our understanding of the impact of music on human emotions and behavior. Advancements in EEG monitoring hardware and recognition algorithm software technology have opened up new avenues for exploring the potential applications of EEG-based music-induced emotion recognition. With these technological improvements, the advantages of personalization, real-time, and convenience in recognizing music-induced emotions through EEG can be further explored in various fields.

At present, the labeling basis of the training set in EEG emotion recognition algorithms largely relies on psychological scales and emotion labels from databases. However, these conventional labeling methods are inherently subjective and discrete. Therefore, there is a pressing need for extensive research to establish a standardized library of emotions based on EEG signals themselves. To address this challenge, musicologists from diverse cultural backgrounds have embarked on initial research into the emotional labeling of music within their respective cultures. As the accuracy of EEG signals for emotion recognition continues to improve, there has been increasing mention of establishing direct EEG signal discrimination for personalized emotion recognition. This advancement holds promise for enhancing our understanding of how individuals from different cultural backgrounds experience and interpret emotions in music, paving the way for more nuanced and culturally sensitive approaches to music emotion recognition (Du et al., 2023).

Improving the accuracy of music-induced emotion recognition can be a challenging problem that demands long-term research, and the advent of deep learning algorithms in recent years has provided a more effective means of addressing this challenge compared to traditional machine learning approaches. With deep learning algorithms, researchers can leverage large amounts of data to train neural networks that can learn to recognize complex patterns and relationships in music-induced emotions. This approach has shown great promise in improving the accuracy of music emotion recognition, allowing researchers to gain deeper insights into how music affects human emotions and behavior. However, ongoing research and development are still required to further refine and optimize these algorithms for use in practical applications (Pandey and Seeja, 2022). As artificial intelligence algorithms continue to undergo continuous optimization and enhancement, new concepts, approaches, and research findings will undoubtedly emerge, offering fresh perspectives and advancing the field of music-induced emotion recognition.

The research and development of music-induced emotion recognition based on EEG relies on the continuous expansion of the application field for such technology. Currently, there are some notable examples of music-induced emotion applications in clinical treatment (Byrns et al., 2020), neuroscience (Luo et al., 2023), and music information retrieval (Li et al., 2023). However, there is still a need for further development of related technical products that can be scaled up and made accessible to the general public, allowing them to better understand and benefit from this technology. This requires ongoing efforts to bridge the gap between research and practical implementation, fostering the creation of user-friendly tools and platforms that can effectively harness the potential of music-induced emotion recognition for broader applications and public engagement. In the realm of consumer applications, there is still much to be explored regarding the potential combination of EEG and personalized music to develop emotional regulation technology and products for users.
